# Systems Modeling at Multiple Levels of Regulation: Linking Systems and Genetic Networks to Spatially Explicit Plant Populations

**DOI:** 10.3390/plants2010016

**Published:** 2013-01-25

**Authors:** James L. Kitchen, Robin G. Allaby

**Affiliations:** School of Life Sciences, University of Warwick, Coventry, CV4 7AL, UK

**Keywords:** population genetics, landscape genetics, spatial individual based modeling, simulation, gene regulatory networks, systems biology

## Abstract

Selection and adaptation of individuals to their underlying environments are highly dynamical processes, encompassing interactions between the individual and its seasonally changing environment, synergistic or antagonistic interactions between individuals and interactions amongst the regulatory genes within the individual. Plants are useful organisms to study within systems modeling because their sedentary nature simplifies interactions between individuals and the environment, and many important plant processes such as germination or flowering are dependent on annual cycles which can be disrupted by climate behavior. Sedentism makes plants relevant candidates for spatially explicit modeling that is tied in with dynamical environments. We propose that in order to fully understand the complexities behind plant adaptation, a system that couples aspects from systems biology with population and landscape genetics is required. A suitable system could be represented by spatially explicit individual-based models where the virtual individuals are located within time-variable heterogeneous environments and contain mutable regulatory gene networks. These networks could directly interact with the environment, and should provide a useful approach to studying plant adaptation.

## 1. Introduction

**Figure 1:**
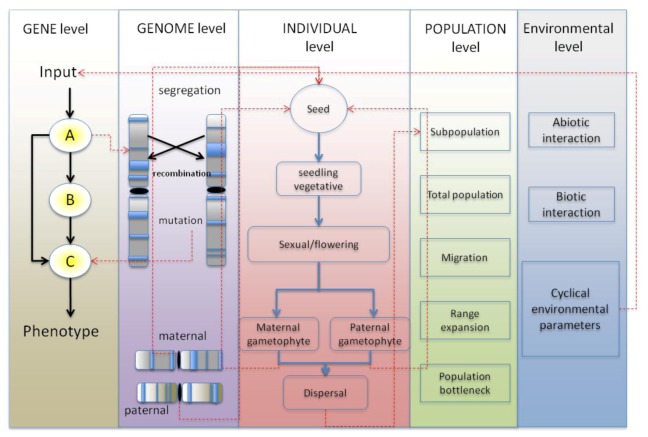
The levels of regulation within the proposed modeling framework. The five levels are: the genic level, genome, individual, population and environment. At the genic level, the genes interact with each other as a gene-regulatory networks (GRN) to produce a phenotype. At the genome level, these genes are arranged into chromosomes, which segregate at meiosis, and the comprising genes mutate and recombine, altering their function. The individual has various life cycle histories and if sufficiently fit from its comprising genetic material, reproduces with other individuals to produce progeny. The individuals make up the populations, which through admix through migration, and can lead to differentiation through bottlenecks and founder effects. The environment contains parameters that change cyclically (or unexpectedly), which is fed into the GRN of the individuals.

There is an increasing awareness of how our climate is changing due to continuing urbanization and industrialization of our planet, and of the possible conservational, ecological and sociological implications. With research studies demonstrating that climate change can affect crops on a genotypic [1] and a phenotypic level [2], it is desirable to improve our understanding for plant adaptation so that it may be exploited to produce crops more resilient to shifting climates, pests and disease, which in turn can be grown to produce larger yields. A related field is the study of genotype-by-environment (GxE) interaction. GxE interactions are widely studied within epidemiological studies [3,4,5], and are of particular relevance to agronomy. These studies are concerned with finding significant correlations between crop genotypes and non-genetic factors, such as climate or environments, in the interests of increasing crop yield [6,7,8,9,10]. In order for such GxE interaction studies to be performed, a comprehensive knowledge of genotypes and of polymorphic non-neutral loci is required. Single-nucleotide polymorphism (SNP) data extracted from using amplified fragment length polymorphisms (AFLPs) are useful markers for demonstrating such genetic differentiation and AFLPs combined with whole genome-scans [11] have previously demonstrated adaptation at different environments such as temperature mediated selection in trees [12]. Mega-bases of sequence data belonging to non-model organisms may now be obtained from next-generation sequencing (NGS) technologies [13] with such studies having been used to demonstrate population differentiation [14,15] and adaptation of individuals to different environments [16,17]. As the environments that organisms reside in are highly dynamic due to regular events such as seasons, night and day cycles, or due to unexpected events such as droughts or floods, it is desirable to quantify the differential gene expression of the alleles of interest. Experimental techniques for accurately identifying protein-DNA interactions such as chromatin-immunoprecipitation coupled with microarrays (ChIP-chip) [18,19] or in more recent years the use of RNA-Seq [20] combined with ChIP-Seq [21] has allowed differential expression patterns at different conditions and the inference of gene-regulatory networks (GRNs) to be determined. Complete GRNs when used in a predictive capacity will provide a useful tool for agronomists to improve crops [22,23]. In recent years, there has also been an interest in merging together the different disciplines in order to assess the differential gene expression data in segregating populations, in the form of eQTL’s [24]. However, despite the numerous mathematical modeling studies that have developed GRNs from expression data, and the numerous programs and tools developed for population and landscape genetics used to model the evolution of individuals with neutral and adaptive loci, to our knowledge no studies have been made to combine the two disciplines and develop models that contain simulated individuals with GRNs that can adapt through a landscape. Such models would be capable of simulating a system that contains the hierarchical levels of regulation that are intricately involved in the adaptation of organisms to their environment. These levels include the gene, genome, individual, population and environment, Figure 1. In such models, a gene may interact with other genes and up-regulate or down-regulate its downstream target genes, eventually inducing a phenotype. The genome accumulates mutations and recombines its comprising homologous chromosomes to produce new genotypic variants, some of which may be adaptive or deleterious. The individual undergoes different life histories, generates gametes, reproduces, and either synergistically or antagonistically interacts with other individuals. The (sub)population collectively adapts to its local environment and may undergo range expansions and admixture with other populations within the meta-population, sometimes outcompeting these populations or forming hybrid zones when speciation events have occurred. Finally, the environment contains dynamic abiotic and biotic factors that may interact with the individuals. Abiotic factors include light or temperature that can change cyclically or unexpectedly, directly impacting on the needs of the individuals (such as facilitating or inhibiting their dispersal for instance), whereas biotic factors from other organisms interact with the individuals of interest. Plants are interesting organisms to model due to their sedentary nature. For example, their dispersal is more limited and often reliant upon environmental features in the case of anemophily and anemochory (wind dispersal of pollen and seeds), hydrophily and hydrochory (water dispersal) or is reliant upon other organisms in the case of entomophily and zoochory, or through cultivation by humans. Unlike animals, they are unable to migrate away from their environments and often exhibit phenotypic plasticity as a result. Furthermore, many plants are allopolyploids and autopolyploids with the potential for providing more of an insight into the underlying genetics, although at a greater complexity. In this review, we discuss previous population and landscape genetics simulation models, including simulations from our laboratory and current methodologies to simulation GRNs. We then move onto examples where population genetics models making use of GRNs will be beneficial within evolutionary biology.

## 2. Current Tools in Evolutionary Biology, Population Genetic and Landscape Genetic Simulation Models

### 2.1. Fisherian Population Genetics Models

Simulation models in population genetics classically are based on a number of simplifying assumptions, such as panmixia, non-overlapping generations and constant population sizes. These assumptions allow the mathematics behind these principles to be described formally and allows the simulated populations to behave in computationally tractable and deterministic ways, such as Hardy-Weinberg equilibria (HWE) [25]. Often these assumptions are biologically reasonable: For instance, it is not uncommon for plant species to be found exhibiting HWE [26,27,28,29,30], especially when pollen dispersal may be distributed via entomophily or hydrophily and seed dispersal via zoochory. Often in these cases, a departure from neutrality can indicate selection. Many population genetics simulation models are based on genealogical trees with many being backwards-in-time coalescent simulations. In coalescent simulations, sampled alleles are traced back via the simulation of gametogenesis until the most recent-common-ancestor (MRCA) has been found [31]. A tree-based forward-time simulation system, TreeSimJ [32], has also been developed, however. Programs such as ms [33] and simCoal [34] are coalescent simulation programs able to simulate genealogies and infer demography and population structure amongst a number of populations. simCoal has three mutation models, a two-allele finite sites model for simulating RFLP data, a stepwise mutation model for microsatellite data and several finite-sites models for simulating mutation of DNA sequence data. The program simCoal has also been further developed to allow for diploid individuals, heterogeneous recombination rates between adjacent loci, multiple coalescent events per generation [35] and to use multiple time points as with ancient DNA data [36]. The program ms has been further developed to process input recombination hotspots [37] and to use elements of a forward time simulator to model selection at a single diploid locus [38].

### 2.2. Landscape Genetics

In reality, geographic landmarks such as lakes, mountains and even roads [39] can provide barriers to gene flow sufficient enough to induce population differentiation, and biotic, climactic and edaphic factors can induce adaptation of individuals at different geographical locations during range expansions. Such biogeographic effects concern the developing fields of Landscape Genetics [40,41], which broadly speaking can be described as a combination of the fields of population genetics and landscape ecology (the field concerned with the interactions between ecological processes and the underlying spatial contexts in which these processes reside). MS and simCoal for example are able to take into account spatial information by the use of migration matrices between subpopulations with either the stepping-stone or island models. Another notable program, SPLATCHE (SPatiaL And Temporal Coalescences in Heterogeneous Environments) [42] along with SPLATCHE2 [43] has been developed in mind to simulate the expansion of a population through an arena comprised of heterogeneous environments. Each SPLATCHE simulation is comprised of two simulations: The first being a forward-in-time simulation of the demographic and spatial expansion, and the second step being a coalescent simulation based on simCoal for reconstructing the genealogies throughout the simulated subpopulations. Here the input terrain files (input from a “vegetation” and a “roughness” *ascii raster* file, the format used in most geographical information systems (GIS)) are used to represent geographic regions with variable carrying capacities and friction values, a parameter used to represent the difficulty of migration from one deme to another. SPLATCHE allows dynamic simulations such that carrying capacities and friction values may change throughout a simulation according to an input file, and can generate DNA, STR, RFLP and standard genetic data as an output. SPLATCHE has been used in previous studies on range expansions [44,45]. A number of other simulation studies have included demic information [46,47] and the use of population units within simulations lends itself conveniently to the calculation of population-based measures of differentiation, such as F_st_. Such simulations could be described as being *spatially implicit* and are often biologically reasonable, as populations can be found within discrete units. For instance Manel *et al.* gives fish in isolated ponds or birds nesting on separate islands within archipelagos as examples [40]. However, many populations are found to exhibit continuous genetic differences across space, as is the case with *Arabidopsis thaliana* over Eurasia and North America [48]. When individuals are distributed across an area exhibiting a gradient of a certain influencing environmental variable, spatial autocorrelations of the genotypes and the variable magnitude can reveal clinal variation: This has been seen with the flowering times of Barley latitudinally across Europe [49]. Such high-resolution genetic data may be obtained by the explicit simulation of individuals rather than populations whose interaction is spatially constrained within a two or a three dimensional arena. Such simulation models are termed *spatially explicit* individual-based models (SIBMs).

### 2.3. Spatially Explicit Individual-Based Models and Their Use in Simulation Studies

Interest in forward-time individual based-models (IBMs) has arisen in the potential for increased individual heterogeneity and stochasticity within the system. Within IBMs, the individual becomes the fundamental modeling unit within the system, unlike mean field models, where populations are represented as homogenous collections of individuals with identical attributes based on summary statistics. The various states that the individual may occupy can therefore be modeled explicitly, allowing for different life histories and other behaviors to be incorporated that may provide more biological realism to the model. These models are generally less efficient than coalescent models, as the coalescent will only simulate genealogies from survived offspring that have made it to the present, and not the entire evolutionary history as with IBMs. However, the greater flexibility posed by forward-simulation models may make them more desirable in some studies and it has been suggested that a tradeoff between the two modeling approaches exists in terms of efficiency and flexibility [50,51]. 

#### 2.3.1. Semi-Spatial Models

A number of software tools using IBMs have been developed. These include EasyPop, a population genetics simulator to simulate neutral loci datasets under various mating schemes and migration models [52]; IBDSim, a program for simulating isolation by distance between individuals [53]; QuantiNemo, an individual-based model for simulating quantitative traits amongst individuals within heterogeneous “patches” [54]; and SimuPop, a flexible simulator that consists of a library of python functions that are required by the user to be “glued together” within a python script, which again has various different mating schemes and migration models at the users disposal [55,56]. GenomePop [57] is an IBM that utilizes Markovian nucleotide or codon models of DNA mutation, such as the Jukes-Cantor or general time reversible mutation model to generate synonymous and non-synonymous mutations. GenomePop thus provides an IBM that can simulate more information at the nucleotide level. GenomePop can also simulate recombination, allow constant or variable population sizes and provides different migration models such as the Island model and the stepping stone model.

#### 2.3.2. Spatially Explicit Models

The programs listed in [42,43,52,53,54] have been described as being semi-spatial [58]. However, due to the flexibility of IBMs they can readily have a fully spatial element incorporated within them to become spatially explicit. Broadly speaking, spatially-explicit individual-based models (SIBMs) contain individuals that are distributed across an area, such as a lattice or matrix (although non-lattice models have been proposed [58]) and may interact with other individuals in a spatially constrained way rather than purely at random. A number of plant-based SIBMs simulation studies have also emerged [59,60,61] in which the spatial element of these models is of particular importance, due to the sedentary nature of plants. The spatial element is of increased importance in anemophilous crops and trees due to their limited dispersal, which follows a “leptokurtic” curve [62]. Doligez *et al. *[59] compared their simulated plant populations, when permitted to form a uniform distribution throughout their matrix, with the clumped populations that readily formed through limited dispersal. They found that the clumped populations exhibited greater spatial genetic structure than the continuously distributed populations, particularly when selfing was allowed. Kitchen and Allaby [60] developed a plant-based SIBM to study the effects of spatial extension between individuals upon the heterozygosity of the plant populations when compared to mean-field HWE expectations. They showed that when plant-mating systems approximated mean-field assumptions (*i.e*., the density was such that the individuals were approximately randomly mating) the observed and expected heterozygosities were largely equivalent. However, the heterozygosity of individuals decreased from mean-field expectations as sparseness amongst individuals increased. AMELIE [61] is a SIBM with a rather more direct application towards food-security and GM crops, and was used to study the amount of introgression from GM forests to conventional forests. It can also allow various life histories and mating systems and can provide demographic and environmental stochasticity. These simulations, however, are only simulating neutral markers and do not attempt to model selection. It is relatively straightforward to take an IBM or SIBM framework and then hard-code a specific adaptive trait, such as one that may influence selection through the perturbation of mortality, or reproductive rate, at a di-allelic or perhaps even a multi-allelic locus if necessary. However, the goal is to be able to account for a possible continuum in the range of landscape heterogeneity and on the strength of the selection inferred from the landscape. One emergent approach is to utilize the concept of resistance surfaces [63,64] and modify the surface in such a way as to produce a “fitness landscape”.

### 2.4. Resistance Surfaces

Resistance surfaces are essentially matrices that contain variables relating to different environmental or landscape features that may impede or facilitate connectivity between individuals in the form of migration or gene flow. They can be parameterized through field data as obtained from GIS systems and are useful for providing hypotheses on the nature of how spatial genetic structure through migration, introgression and dispersal may have formed. One notable SIBM that utilizes resistance surfaces is CD-POP (Cost Distance POPulations) that contains cost distance matrices for representing resistance to movement through the landscape [65]. The program uses gradients of cumulative cost to impede dispersal between grid cells and can facilitate reproduction according to four different functions: linear, inverse square, nearest neighbor and random mixing. The initial version of CD-POP could be used only with neutral loci. However, this was improved upon in an important follow-up paper where CD-POP made use of a fitness landscape in order to simulate selection [66]. CD-POP was upgraded to include a di-allelic single or multi-locus system with any number of neutral loci and up to two unlinked, di-allelic, selective loci (with alleles A, a, B, and b). Selection is then implemented according to the grid value where generated offspring reside and the genotypes of the selective loci that they contain. This represents an important step towards providing a general model for simulating selection. More recently, another study utilizing CD-POP’s selection model has been used in a study to assess the role of adaptive and neutral markers towards population differentiation [67]. Another open-source software tool that uses resistance surfaces is Circuitscape, which is based upon resistance paths that are analogous to those within an electrical circuit [68]. It may be used to predict dispersal of animals or plants and patterns of genetic differentiation among in heterogeneous landscapes [69].

These efforts in landscape genetics simulations represent the first stages into relating genotype to environment and the resulting effects on selection and adaptation. As with CD-POP, different genotypes of unlinked loci may produce different effects on fitness of an individual according to the spatial grid point on which it is located. However, in reality genes do not exist in isolation but exist in networks, and through cis-acting and trans-acting regulatory effects can up-regulate or de-regulate each other, ultimately affecting the expressed phenotype in a dynamic way. It has therefore been suggested that in the interest of genotype to phenotype mapping, genes should be considered in the context of networks [70]. We discuss genes within networks in the next section.

## 3. GRNs, Network Motifs and Inference

**Figure 2:**
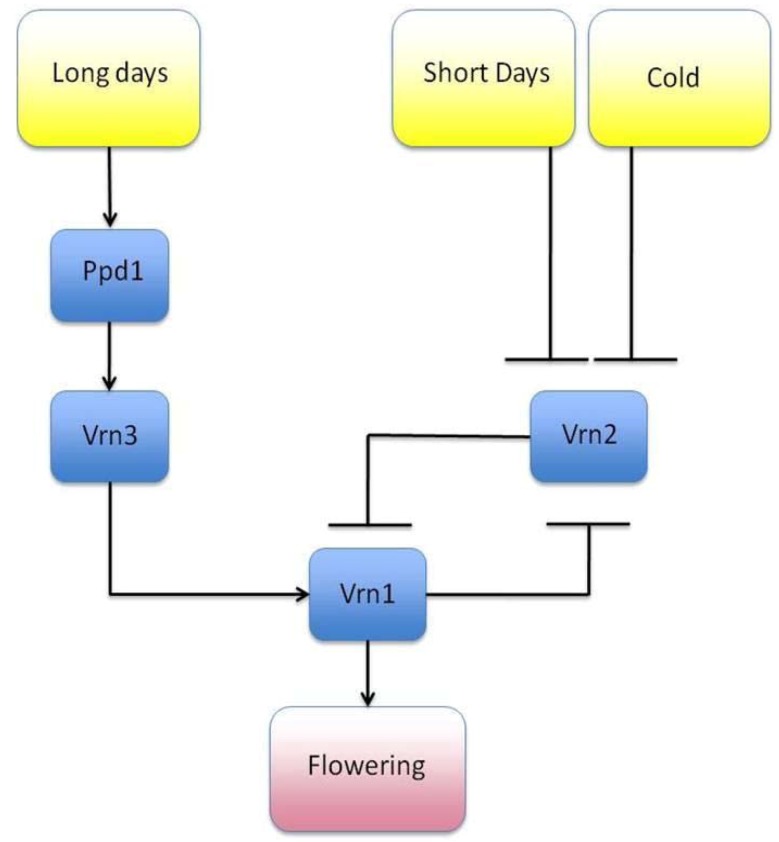
Vernalization and photoperiodicity in Barley. Gene Vrn2 negatively down-regulates gene Vrn1, preventing flowering. During periods of cold, short days, Vrn2 is down-regulated. However a period of long days is required to activate genes Ppd1 and Vrn2, which activate flowering.

Efforts to ascertain all the interacting genes with regards to the expression of a particular phenotype is an area of which is highly relevant to most, if not all, disciplines within biology. Such information, for instance, could provide biologists with potential molecular targets, be they genes, proteins or metabolites, whose function may be altered through gene silencing, catabolism, or through agonistic or antagonistic ligands. The identification of GRNs has multiple uses ranging from developing drug targets in complex disease, understanding stress response (with clear uses in developing drug targets and in agronomy), decreasing antibiotic, herbicide or pesticide resistance and identifying key developmental genes. One application of a GRN can be to model transcriptional networks within a cell, although interactions at the proteomic and metabolomic level and other areas of the “interactome” may also be modeled. Transcription factors (TFs) may behave as transcriptional activators that up-regulate other TFs or behave as transcriptional repressors that can down-regulate their targets. The crosstalk between the up- and down-regulation of transcription allows dynamicity to the amount of protein product that is expressed, which ultimately, will have an effect on the phenotype of the individual. One example is the GRN regarding photoperiodicity and vernalization of barley as described by Fuller and Allaby [71] (Figure 2), which is closely related to the GRNs of wheat and *Arabidopsis thaliana*, a model organism widely used in GRN related studies [22]. In this, relatively simple, pathway, gene Vrn2 down-regulates Vrn1, which through a series of upstream interactions indirectly promotes flowering. The increased cold and lower amount of light from the shorter days in winter down-regulates Vrn2, thus limiting the repressive effect Vrn2 has upon Vrn3 and Vrn1. This lack of repression is insufficient, however, to promote flowering alone and a period of long days during summer is required to activate gene Ppd1 and the remaining cascade, which leads to flowering. This simple example emphasizes the role that cyclical environmental patterns have upon expression of the phenotype. Indeed, through mutation the sensitivity of these genes to their environmental inputs may become altered. For example, a loss of function mutation of PPD1 renders the plant less sensitive to sunlight and delays flowering, whereas a loss of function mutation in VRN1, VRN2 or VRN3 results in an early flowering phenotype due to increased sensitivity. These mutations have been shown to be the cause for clinal variation of Barley across Europe [49], with late flowering plants being more prevalent in darker northern Europe, and the early flowering phenotype more common in southern Europe.

### 3.1. The GRN Topologies Observed in Nature

**Figure 3:**
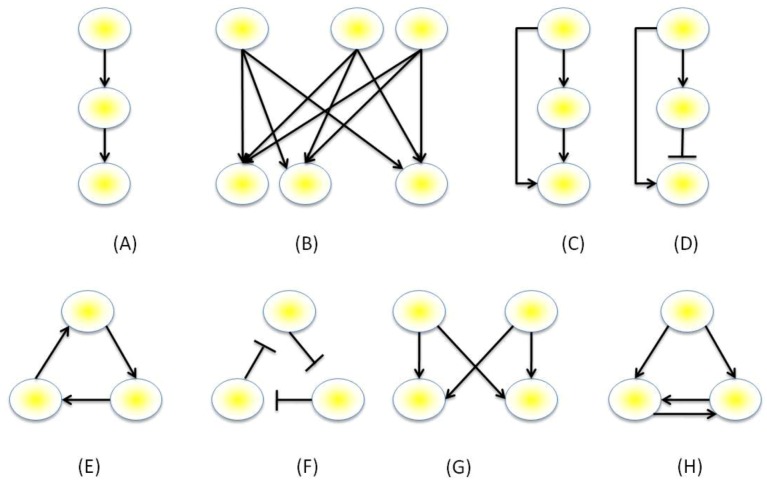
Example network motifs. (**A**) Single input module (**B**), multi-input module, (**C**) coherent feed-forward loop: The motif consists of a direct and an indirect pathway to activate the final gene. (**D**) Incoherent feed-forward loop: The overall sign of the indirect and direct paths differ. (**E**) Three-cycle positive feedback loop, (**F**) three-cycle negative feedback loop, (**F**) bi-fan motif, (**G**) double-positive feedback loop.

The genes within a network may be visualized as directed graphs containing a set of nodes, representing the genes, protein and/or metabolites, connected by a set of edges, which represent the interactions between these nodes. The number of edges that belongs to a node is its degree, and the distribution of the number of edges across networks is the degree distribution. Intuitively it may be assumed that the degree distribution would approximate a Poisson distribution, however, conversely they tend to approximate a power-law distribution, where most nodes are sparsely connected and a small number has a much larger degree [72,73]. When auto-regulation of genes is not permitted, the maximum number of edges within a network of size N must necessarily be N(N-1) edges, however, many genes do regulate themselves as in single-gene positive or negative feedback loops. Expression data obtained from technologies such as Yeast 2-Hybrid, ChIP-chip or ChIP-Seq can provide relationships such as correlative relationships between sets of expression data. The resultant expression data can be processed by software and mathematical models can be inferred (reviewed in [74,75]). An interesting paradigm emergent from this data is the existence of common network topologies that are observed across different taxa and even different types of networks (*i.e*., non-GRNs). This paradigm was first observed by Milo *et al.* [76,77] who generated null distributions of network sub-graphs through randomizing the edges of networks with the same degrees, and selected motifs that were found to be in numbers significantly higher than at random [76]. A follow up study used z-scores to calculate a significance profile for comparison of network local structure when compared with random structures [77]. Both studies found commonly occurring motifs not only within transcriptional networks, but also within protein-signaling networks, neuronal networks and non-biological networks, such as those found in social networks, power-grids and within the World Wide Web. These methods did receive some criticism, however. For example, it was stated that *C. elegans* neuronal pathways are spatially dependent with networks being formed between spatially closer nodes and that these spatial dependencies were not included by Milo *et al.* in their network inference [78]. Common examples of the motifs observed are illustrated in Figure 3. These include the single and multi-input modules, the positive feedback loop, the negative feedback loop, the three-cycle positive feedback loop, the feed-forward loop (FFL) and the bi-fan motif.

### 3.2. Motif Function

Putative functions of these motifs illustrated in Figure 3 have been described by Alon [76], and it has been suggested that certain motifs can facilitate one of two roles: Either as sensory networks, which respond to nutrient levels and facilitate stress responses; or memory-based networks, which act as irreversible switches with putative roles in organism development or cellular differentiation. The FFL is an extremely common motif and has been shown to have either coherent (where the sign of the direct pathway equals the overall sign of the indirect pathway) or incoherent (the signs of the two paths differ) behavior, Figure 3. The coherent type-1 FFL has been shown in studies using *E. coli* to be a “sign-sensitive delay” element and a “persistence detector” [79,80]: For example, when both paths need to be active for activation of the final gene in the pathway (“AND” behavior), time is required for transcripts of the intermediate gene in the indirect pathway to accumulate sufficiently to become active, delaying up-regulation of the final gene. Conversely, when either pathway is sufficient to activate the final gene (“OR” behavior), up-regulation by the intermediate gene of the indirect pathway of the final gene will persist, even after the initial gene in the pathway has been deactivated. The incoherent type-1 FFL has been described as a pulse-generator, with such behavior observed in *E. coli* [81]. Here, activation of the initial gene will immediately activate the final gene. However, once the indirect pathway’s intermediate gene is activated, transcription of the final gene is halted, generating the pulse. An example of a memory-based motif is the double-positive feedback loop motif, Figure 3. In this motif, activation of the top gene will activate both of its target genes. The reciprocity amongst these two genes will keep them locked into being constantly activated even when the top gene is no longer activated, hence, they retain a “memory” of having been activated. This sort of behavior would be appropriate for irreversible processes that can decide the fate of a cell, such as differentiation, reproduction or apoptosis.

The apparent commonness of many of these motifs has attracted much attention, and a number of studies have been made to help explain this paradigm. One explanation is that these motifs are dynamically stable and are robust to small perturbations in signal [82], and that this robustness could account for the motif’s apparent abundance. Mutational robustness or insensitivity of the genes within the motif to mutations, could also lead to an abundance of these motifs in nature. However, Widder *et al. *[83] recently studied the kurtosis of the probability distributions for the FFL to perform a range of different functions and computationally studied the effects of repeated mutations to the functional robustness of the motifs. Their results suggested that the abundance is more influenced by the plasticity of the FFL in performing a wide-range of functions and that mutational insensitivity was unlikely to account for the abundance. A wide range in function of the Bi-fan motif has also been reported [84], with a caution from the authors of the study that the particular structure of a motif should not necessarily be expected to guarantee a particular function. Furthermore, a study by Konagurthu and Lesk [85] reported that through their implementation of a random-edge search algorithm, the frequencies of common motifs within natural networks was similar to those within random networks. They also noted that random connectivity within a three-node network, such as the FFL loop or a three-member positive feedback loop (3-cyc) would naturally form an FFL due to the search space involved (with 2^3^ possible conformations, six will be consistent with FFL architecture, and two with the 3-cyc) and that the search space may account more for the abundance than the function.

### 3.3. Mathematical Modeling of GRNs

Developing GRNs from experimental data is often described as reverse engineering, or network inference, and comprises a particularly large field within the discipline of systems biology. Although major advances in experimental techniques and advances in modern computing power have no doubt assisted efforts in network inference, it still remains a non-trivial task. Ultimately the quality of an inferred network model is highly dependent upon the quality of the data, and this can come at a considerable cost with large networks, as the amount of required data is proportional to the number of network nodes. Perturbation experiments such as generating gene knock-outs, stress experiments or RNAi experiments can provide an informative insight into the dynamicity of a particular network. However, the large amount of noise within expression data often requires that experiments be repeated in order to determine the extent of the noise. Constraints on the GRN can be placed to alleviate the model’s complexity and data requirements, however. These include limits on the number of nodes in the inferred network (thereby generating a sparser network) and restricting the model parameters, e.g., through connectivity limitations. It is also often desirable when inferring a network to make use of prior biological knowledge (such as molecule binding sequence motifs, posttranslational modification sites or molecular interactions), which may assist with model validation or with constraining the model complexity. A number of online repositories of such information are available such as the Gene Ontology (GO) or the Kyoto encyclopedia of genes and genomes (KEGG).

#### 3.3.1. Boolean Networks

**Figure 4:**
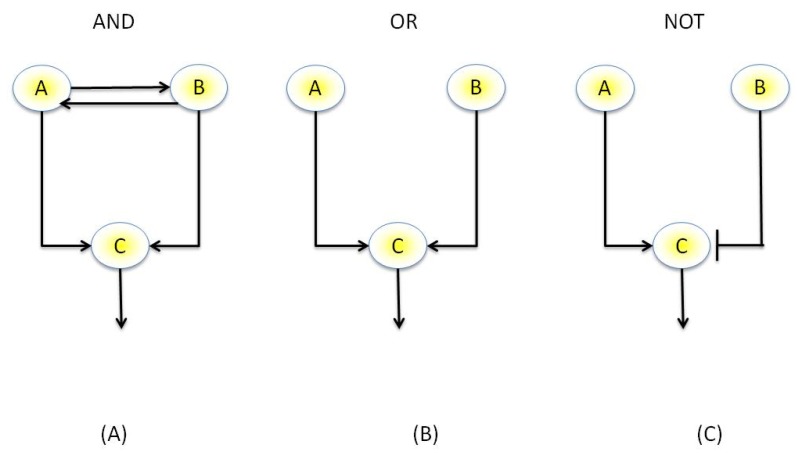
Example of a Boolean network. (**A**) AND motif: Genes A and B co-regulate each other, therefore Genes A and B must be active to activate Gene C (**B**) OR motif, either Gene A or B is sufficient to activate Gene C (**C**) NOT motif: Gene B down-regulates Gene C, therefore must be “off” to allow activation of Gene C.

The activation of some genes within a network may hold certain dependencies with the activities of other genes, such as the “AND” and “OR” behavior described previously. Thus, it is possible to represent genes in a similar manner to logic gates, where a gene may belong to one of two discrete states, namely “ON” or “OFF” and hold a set of discrete dependencies in terms of activation with other genes in the network, such as “AND”, “OR” and “NOT” relationships, Figure 4. Boolean representations of genes were first described by Kauffman [86] and are widely used today. An example piece of software for inference of Boolean network from experimental data is REVEAL (REVerse Engineering Algorithm) [87], which enumerates through all possible Boolean networks from the input data and uses mutual information to score each network, with the most sparse network that best describes the data being given as the optimal network. Boolean networks, which although able to represent dynamical networks, do have quite clear limitations, however. Firstly the transcriptional levels of a gene are continuous values and cannot simply be discretized into a binary variable such as “on” or “off”. Multiple discrete states, however, such as “gene product present” or “gene product absent” as well as “on” or “off” have been proposed [88]. Furthermore, Boolean networks are intrinsically deterministic and may be inadequate for describing the various stochastic effects within a network. To this end, probabilistic [89] and more recently stochastic [90] Boolean network variants have been proposed, which retain the rule-based determinism of Boolean networks yet can better model uncertainty.

#### 3.3.2. Continuous GRN Models and Bayesian Networks

Genes are not simply active or inactive, yet are transcribed at continuous rates so that the amount of transcript for one gene is dependent upon the rate of transcription of another gene (although this may be discretized through the use of “gene thresholds” for activation in modeling efforts). This lends itself conveniently to using ordinary differential equations (ODE’s) to represent GRNs. The resulting modeling functions used may be linear or non-linear with an example software tool used to infer linear models from expression data being EXAMINE (Expression Array MINing Engine) [91]. Another approach for the mathematical modeling of GRNs is to describe gene expression values as random variables following probability distributions, such as in Bayesian inference [92]. Bayesian networks form a directed-acyclic graph (DAG) and may represent dynamic or static (*i.e*., representing a GRN once a steady-state has been reached) networks using continuous or discrete data, and are readily able to model the randomness and stochastic effects that may exist amongst GRNs. This makes them more robust in the presence of noise or missing data than Boolean networks. Another benefit of Bayesian networks is that they provide a framework that allows researchers to incorporate prior knowledge for network inference. However, caution should be made when little or no information is available, as the use of uninformative priors (e.g., uniform priors) can make Bayesian network inference inefficient. As Bayesian networks are formed with a DAG, static networks cannot represent cycles such as in feedback loops. However, this limitation is not present with dynamic Bayesian networks [93], as they avoid cyclical representations by using discrete time steps to separate input nodes (e.g., at time *t*) from output nodes (e.g., at time *t *+ ∆*t*). BANJO (BAyesian Networks with Java Objects) is a software tool that has been developed for the inference of static and dynamic Bayesian networks [94].

## 4. Synthesis: Spatial Individual-Based Models with Gene Networks: Approaches, Applications to Plant Science and Potential Pitfalls

Within this review we have discussed theory within the fields of population and landscape genetics and systems biology, and have described software and approaches to simulating adaptation. We believe that modeling efforts within evolutionary biology have reached a suitable step where coupling systems of genes to SIBMs that can interact with the surrounding environment and induce phenotype in a more complex and perhaps more biologically reasonable way, can now be considered. Ultimately, a unified approach based upon stochastic elements of GRN evolution, migration and range expansion could allow emergent paradigms in how phenotype relates to GRN topology and raise questions as to how this relates to different abiotic and biotic interactions at different spatial locations. Thus, such systems could direct research into a number of previously unanswered questions in evolutionary biology and evolutionary systems biology, including:

1. How does a functional (non-neutral) mutation to the sensitivity (as in threshold) or output of a GRN node affect the expressed phenotype or the fitness of an individual? How do the phenotypic effects differ from simulating single non-interacting loci?
2. How do perturbations of the edges within a network (such as edge deletion, addition and rewiring) or node duplications impact on the fitness of an individual within different environments?
3. How does the conformation of a GRN affect the quantitative trait that is ultimately expressed? Can population models or SIBMs show that certain motifs may be selected for within different environments?
4. What role do evolutionary forces such as gene flow and range expansion play on the diversity of GRN topologies?
5. Considering the effects of gene flow, can certain environments (*i.e*., abiotic factors) favor specific GRN topologies? Similarly, can biotic interaction select for certain GRN topologies?
6. Which choice of GRN representations (such as static-edge, Boolean, Bayesian, ODE-based networks) is a better fit to the system in question?

The first two questions require the use of GRNs, whereas the last two require a spatial element and a landscape genetics approach to provide sufficient environmental heterogeneity. We discuss these elements in the next two subsections.

### 4.1. GRN Evolution and the Resulting Phenotypic Effects

#### 4.1.1. Simulating GRNs in Population Models Instead of Quantitative Trait Loci

GRNs have so far received little attention within evolutionary studies at the population genetics level [95]. Studies of genotype by phenotype interaction commonly involve the analysis of quantitative traits, such as seed size or petal color that are influenced by one or more loci. Therefore the modeling of quantitative traits or even quantitative trait loci (QTLs) may be a viable alternative to explicitly modeling GRNs and may benefit a model in terms of efficiency or when there is insufficient data in which to infer a GRN. However, QTLs themselves may interact with cis-acting or trans-acting elements on the transcriptomic and proteomic levels, and may code for catalytic proteins that interact with substrates on the metabolomic level, before the quantitative trait is expressed. It has also been suggested that all genes are not equivalent regarding their evolutionary role, as in standard population genetics models, yet it is a gene’s position within a network that determines its evolutionary role [96,97,98]. Therefore differential effects on phenotypic variation may arise from mutation of the genes in a network. For instance, we have already discussed an example found in nature with mutation of the nodes within the photoperiodicity system (Figure 2) causing either late or early flowering times. Allelic variants of these elements may also be under selection: For example, we know that 6% of the human genome is currently under selection, yet only 1.5% of the genome is protein coding [99], with the rest of the purifying selection possibly on regulatory elements. If selection favors co-inheritence of a collection of alleles which interact with each other within a GRN, then these alleles may also be placed under linkage disequilibrium and not become segregated by recombination [100]. Therefore simulation of GRNs may provide researchers with a better understanding of the specific alleles that need to be in a network to fully take advantage of a given set of environmental conditions.

#### 4.1.2. Simulating Network Evolution

Evolution in the context of GRNs has been receiving more interest in recent years [96,101], especially in the field of evolutionary developmental biology [102,103] or Evo-Devo, concerned with the comparative analysis of the developmental processes of species and of the evolutionary relationship between the developmental processes. The bioinformatics community is also becoming increasingly interested with the study of the ancestral relationships between biomolecular networks, with algorithms being developed for network alignment [104,105]. In their review, Knight and Pinney [101] describe seven mechanistic perturbations of biological networks including rewiring, or new edges being introduced between nodes; node duplication; node loss and entire network duplication. It has been shown that a single point mutation is sufficient to induce entire proteomic network rewiring [106]. It is also understood that duplication may lead to sub- and neo-functionalization within networks, where either the resulting paralogs take on separate functions from each other (where the ancestral gene was capable of all functions) or one paralog takes on a new function, respectively. The concept that single gene and whole genome duplication could lead to evolutionary diversification has existed for decades [107] and is still commonly under study [108,109]. We have already discussed the widely documented examples of network motifs found within biological motifs, their potential roles and how their structure may relate to function, if at all. Whether the structure of a motif necessarily relates to function may currently be a topic of debate, however, it is conceivable that selection for a particular phenotype may require a specific structural motif, and this has been suggested for the positive feedback loop [110]. There have also been a number of studies of how motif structure may influence stochastic fluctuations, or noise, from a network motif, and it has been suggested that noise itself can be placed under selection [111]. Noise may control organism stress-responses such as persistence in bacteria, where the cell may enter a state of dormancy in harsh environmental conditions at the cost of cellular growth rate. Through mathematical modeling of the HipBA toxin-antitoxin system in *E. coli* [112] Koh and Dunlop showed that by altering the architecture of the network (through removing feedback and placing the two genes on separate operons), they were able to alter the frequency of persistence, a trait that could be selected for in different environmental conditions [113]. Interestingly, a study from Tsong *et al. * [114] demonstrated that for the two species *S. cerevisiae *and *C. albicans*, a particular network shared by the two species had been reversed in structure (one regulated by a repressor, the other by an activator). The “logical output” or phenotype, however, remained the same due to several changes in cis- and trans-regulatory elements. Therefore network evolution may converge to the same outcome as well as diverge.

#### 4.1.3. Choice of GRN Model within the Context of a Spatially Explicit Individual-Based Model

The GRN reverse engineering approaches described in Section 3.2 can be conceptualized as “top-down” processes, where we begin with a phenotype of an individual (*i.e*., after subjected to stress or after a gene knock-out procedure), observe the expression patterns, and infer a genetic model from the data using statistical and mathematical approaches. However, the inferred networks and the modeling paradigm used to describe it (such as Boolean or continuous GRNs) could readily be used in a “bottom-up” approach to demonstrate the range in expression and/or the resulting phenotype once subjected to different environmental inputs. We therefore believe that SIBMs parameterized with resistance surfaces or landscape patches provide an excellent framework for producing such models. The GRN could be represented using a Boolean network form or as a continuous form, using linear or non-linear ODEs, that would take its input from the surrounding environment, interact with the other nodes in the network and produce a phenotype. Gene threshold parameters could be used to define the criteria needed for activation, and genes at the top of the network could directly interact with the environment. Whereas Boolean or ODE-based GRNs would classically represent deterministic networks, the output on each gene could instead be a random variable generated from a certain probability distribution, providing a network that may more approximate Bayesian networks. A potentially interesting study could be: If given genetic network data within a real environmental system (such as the distribution of flowering times latitudinally across Europe), which GRN model best explains the data and provides the maximum likelihood?

### 4.2. Benefit through Using a Spatially-Explicit System

In this review we propose that research should be directed towards looking at the phenotypic effects of network evolution in the context of populations located within patchy landscapes. The addition of spatially explicit heterogeneous landscapes will add another layer of complexity to any model, and adding any intra-annual variation in environmental parameters will increase this complexity. Although it is not the goal of modeling to accurately represent nature in all of its complexity, we argue that such extra detail is necessary in order to fully understand how phenotypic variation (through mutation of GRNs) may emerge and become selected for or against. Firstly we need to adequately model gene flow, which provides the homogenizing force between subpopulations that would otherwise ultimately differentiate through a process of mutation and genetic drift. Although the flow of chromosomes containing genes that may interact with one-another in a GRN may be modeled within a mean-field system, gene flow itself is often spatially constrained and may be influenced through geographic landmarks, such as mountains, rivers or roads. Impeding gene flow can lead to increased population differentiation, which can lead towards speciation. The explicit modeling of space is a convenient way to allow the simulation of range expansions and the subsequent limiting effects on allelic diversity through the subsequent founder effects. Incorporating heterogeneous environments into the spatially explicit arena will also allow abiotic interaction to select for different alleles, and possibly select for different GRN conformations. For example, the GRN conformations for Barley, wheat and *Arabidopsis* have shown to be quite different, despite sharing many of the same components [71,115]. A particularly fundamental question to be addressed in evolutionary systems biology is why do certain GRN conformations exist in different environments and why are they favored in some way? One possible way to answer such a question could be to keep GRN topologies constant and randomize environmental parameters according to a given prior distributions, as in a Bayesian analysis.

#### 4.2.1. Biotic Interaction

We have described how the resistance surfaces that may be explicitly incorporated into an SIBM may represent climactic or edaphic factors that can impede dispersal or influence selection of the simulated individuals. However, in a similar vein, they may also represent biotic interactions from animals or plants. Biodiversity varies latitudinally across the globe [116], and biotic interaction is thought to be of particular importance in the tropics [117]. One example of biotic interaction is seed predation, and this has famously been proposed in what is collectively termed the Janzen-Connel hypothesis [118,119] to prevent competitive exclusion. Seed predation can be represented in simulations as probabilities of predation for dispersed seeds, either throughout the entirety of the simulation or at individual grid-points, for example. It may be difficult in this approach, however, to simulate the dynamics of predator-prey co-evolution, unless some form of dependency was incorporated between the modeled individuals and the resistance surfaces. Another approach is to have multiple classes of individuals within a simulation that could represent “species”. Individuals belonging to different species could then be modeled with different GRNs, as has been seen in nature with the barley, wheat and the *Arabadopsis* photoperidocity network. Individuals may then compete for space (in order to germinate). If the model is specific and growth and nutrient uptake are explicitly modeled (see Section 4.6.2 on functional-structural plant modeling), then different species could potentially compete for resources.

#### 4.2.2. Analyzing Past and Future Events on Adaptation

GRNs are dynamic, and therewith comes the necessity of incorporating time-dependent environmental variation when GRNs are simulated within the context of SIBMs. A natural extension of this is that it will become convenient to study past shifts in the environment onto the genotypic and phenotypic characteristics of a population (such as through the effects of bottlenecks and migration, for example). Hypothesized future effects could also be studied in a similar manner.

### 4.3. Producing Complex Modeling Systems in a Step-Wise Manner

Complicated models with multiple levels of regulation could be developed within a step-wise manner, yet there is no one correct path a researcher may take. The model should be validated as each level of regulation (Figure 1) is added. Deterministic systems based on mean-field assumptions such as Hardy-Weinberg equilibria may provide a means of model validation. Complex models may require time-consuming simulations, and if there is much stochasticity in the system, it could become difficult to interpret their results. Therefore a suitable strategy might be to start with simple models, such as mean-field models and/or single population models. For example, the initial stage of a modeling study could be to begin with a population of limited spatial structure, single genes or QTLs and only neutral non-selective abiotic parameters, where the only source of genetic variation is through mutation and genetic drift. After validation, extra elements could be added including a more heterogenous environment and a rudimentary GRN, and so on. If a modeling system is designed in order to be modular, as in to allow certain features to be enabled or disabled in the model, it may be convenient to begin with simple systems and prevent the need to develop new models for each step of the study. 

The relevant question here is at which level of regulation the modeler begins, which will be highly influenced by the hypothesis that the researcher intends to address. One possible hypothesis could be that certain environmental parameters would select for a particular GRN variant, for example, and so a study might involve analyzing the effects of GRN conformation on individual fitness. GRN conformation could indicate the shape of its degree distribution, or could simply mean choice of structural motif, for example. In the first study, simulations could provide data on fitness (in the form of population growth curves, for example) for different GRN configurations that are kept constant (*i.e*., no mutation or rewiring) throughout the simulation. In a subsequent step GRN reconfiguration could be enabled and the final configuration recorded, to determine whether GRNs have evolved into an “optimal” configuration. Final simulations could involve allowing populations containing evolving GRNs expanding throughout a heterogeneous landscape, and spatial genetic structure could be analyzed. 

Another study might be to attempt to explain the spatial genetic structure of a population found in nature, for which GRN data exists, through attempting to recreate data observed in nature (such as allele frequencies or selection coefficients). Initial simulations could be within mean-field systems, with non-stochastic migration rates between subpopulations and only single gene nodes or QTLs being simulated. Subsequent simulations could add spatial explicitness, abiotic and biotic factors and GRNs. At each step of the study, likelihood densities could be generated to explain which models best explain the observed data. Our research group has previously applied Approximate Bayesian Computation (ABC) [120] to our SIBM in our research (currently unpublished). ABC can be a powerful numerical technique within population genetics. It allows for likelihood densities to be generated from parameter subsets that can simulate summary statistic data that is sufficiently close to data observed in reality. It has been widely used within a number of population genetics studies thus far (for example, see [121,122]).

### 4.4. Adaptive Dynamics

When simulating selection in models it is important to consider the role of evolutionary tradeoffs and how they may influence the adaptation of a species. Antagonistic pleiotropic effects [123,124,125] as first proposed by Williams [123] occur when a mutation with a beneficial change in fitness on one trait has a detrimental effect upon another trait. This can lead to the emergence of evolutionary fitness costs [126,127,128,129,130,131,132,133] where increased resource allocation from one function leaves more limited allocation to another function. One plant example of a trade-offs in the literature is increased transposable element silencing despite deleterious effects on the expression of nearby genes [129] in *Arabidopsis thaliana*. Another study showed that increased investment in female and male reproductive structures limited the quantity and nitrogen content of clonal propagules, respectively, in *Sagittaria* *latifolia* [126]. A further example exists in *Arabidopsis thaliana* where a mutation in the EMBRYONIC FLOWER (EMF) genes EMF1 and EMF2 induces very early flowering but also a reduction in seed production [134]. Thus evolutionarily “perfect” organisms are not trivial to obtain. Trade-offs may also exist according to the ecological characteristics within the geographical area that a population resides within. To give an example: Selection for increased plant size may increase the rate of depletion of the nutrient resource within the soil, thus, adaptation of the plant population to its surrounding environment in turn influences the environment. In order to help study such dynamic genotype by environment interactions, the 1990s saw the emergence of the field of adaptive dynamics (AD, reviewed in [135,136]), which, through mathematical modeling allowed the researcher to gain an insight into the long-term dynamics of the evolutionary and the ecological processes within a given system. AD has developed from evolutionary game theory and the study of evolutionary stable strategies, which may describe the payoffs associated with a mutant, *m*, of strategy A invading a resident population, *r*, with strategy B. It makes four assumptions: clonal reproduction, separation of ecological time scales, small mutational steps and a small initial invading mutant frequency within the monomorphic population *r*. The invasion fitness is given as the exponential growth rate of a *m* within *r*. Positive values of *f* indicate that *m* will successfully invade and replace *r*, and negative values indicate that the mutant will be unsuccessful in invading the resident. Using the invasion fitness function, *f*, pairwise invasion plots (PIPs) may be plotted. PIPs are two-dimensional plots where the zero contour line is plotted at the various quantitative values of the *m* and *r* phenotype, allowing potential regions of invasion success and failure to graphically be identified. Intersection of the isocline at the 45-degree line from the origin (where *m *= *r*) allows identification of possible evolutionary end points at certain values of the resident phenotype. Using the AD framework, Geritz *et al.* [137] produced a model to study the evolutionary dynamics of seed size, which contained a trade-off between seed size and seed number. They were able to adjust the influence of the seed size on the competitive ability of their seeds (which they called competitive asymmetry), the resources per germination site and the type of precompetitive environment in which their seeds resided (a continuum from favorable to unfavorable). They found that strong competitive asymmetry, high resource levels, and intermediate harshness of the precompetitive environment favored a polymorphic population containing the coexistence of plants with different seed sizes, where although a single large seed may outcompete a single small seed, the higher numbers of smaller seeds was also competitive. Boudsocq *et al.* [138] presented an AD study that investigated the trade-off between plant size (due to increased nutrient uptake), where larger plants are fitter, and increased plant mortality with greater nutrient uptake. The authors set out to determine whether natural selection could lead to “evolutionary suicide” or Harman’s “tragedy of the commons” where resources become too depleted to allow plant survival, or whether Tilman’s R* rule, where the plant with the lowest steady-state resource level is selected for will apply. In their model, Boudsocq *et al.* found that evolution leads to a minimization of soil mineral nutrient content, yet the nutrient resource was not intensely depleted, supporting Tilman’s R* rule.

#### Simulation of Evolutionary Tradeoffs with GRNs

Such example AD studies have the benefit of allowing researchers to quantify the effects of certain trade-offs to an evolutionary system. We believe the modeling framework proposed in this study of coupling GRNs to SIBMs could also allow for such tradeoffs through interactions between one gene and numerous target genes/traits. When considering complex interconnected networks, it becomes clear that potential trade-offs could be programmed into the system. For instance, a simple example may be where mutation of a gene may cause up-regulation of one or more of its target genes with beneficial fitness effects to trait A, whilst this may indirectly negatively impact the fitness provided by trait B. However, as with the AD framework, such interactions have to be hypothesized. This may not be the case, however, if a model is complicated enough to allow for GRN re-wiring. Through stochastic GRN re-wiring through mutation and movement through a heterogeneous landscape, emergent trade-offs may be observed that may not have previously been hypothesized. This may provide opportunities to document such trade-offs and analyze their evolutionary impact.

### 4.5. Pitfalls

#### 4.5.1. Algorithmic and Programming Complexity

The complexity of SIBMs is not trivial and development of a large simulation software tool may not be without problems if inadequate care is not put into the development process, or if there is ambiguity in its function, as this may make the tool difficult to communicate or reproduce. To this end a few authors have proposed protocols that can be used in the design and development of IBMs [51,139]. SIBMs are generally less efficient than aspatial IBMs due to the processing of spatial distances or landscape values, if landscape information is incorporated. The use of a quadtree structure [140], which breaks the two-dimensional space down into nodes and are stored in a hierarchical way (as in a tree-like data structure) may provide some optimization over brute-force searches when individuals interact over space. A further approach for optimization in landscape genetics based on the quadtree was to use a hierarchical system of patches within an irregular grid [141]. Although the efficiency of developed software tools poses one problem, the implementation of complex systems within a model can be non-trivial, especially if interacting genes and environmental information are to be incorporated. Software engineering approaches [142] into the design of a system provide a more thoroughly planned design-process that will allow a greater transparency of the system specification to non-developers and may prevent design flaws or other complications during the development phase. These include the use of process-management models including the waterfall or iterative model, analysis and design of behavior using data flow diagrams, and the use of diagrams specified within the unified modeling language (UML) such as class hierarchy diagrams for object design and use-case diagrams for system interaction analysis and design. Object-oriented programming languages, including languages such as C++, Java, C# and Python provide a number of concepts such as object-inheritance, polymorphism, abstraction and interfaces, which can greatly facilitate the design and implementation of IBMs. For example, classes such as Individual, Gene, Genome, Chromosome and Patch could be implemented, and a number of individual-based modeling studies have taken similar object-oriented approaches [54,55,56,60,141,143]. However, it has been suggested that the use of certain features within SIBMs, such as environmental or terrain features, may be best not represented as objects [144]. Furthermore, the implementation of an IBM using an object-oriented approach in Java and C++ was shown to be less efficient than when implemented with a procedural approach in Fortran 95 [145]. Inexorably, object generation can be computationally costly, therefore, excessive use of objects when unnecessary should be cautioned against.

#### 4.5.2. Accurate Representations of GRNs

Arguably the most obvious pitfall with using such models is the high computational cost associated with the large ranges in scale required, from subcellular processes within the simulated individuals to the dynamical environment in which they reside. It is generally required that SIBM simulations be run with thousands of individuals, therefore, large GRNs with large numbers of nodes and large numbers of edges may become more intractable. Furthermore, sensory based GRNs such as the delay-response element and the persistence detector mentioned in Section 3.1 may become difficult to implement within simulated individuals as they represent time-dependent processes at a microscopic-scale, with a requirement for continuous transcript levels that builds up or breaks down over a period of time. The level of detail required for such processes could greatly slow down the rest of the simulation at the individual, population and environmental scales. If the simulation model was also run using discrete time steps (such as generations or months), a particularly fine-grained time step, such as hours or even minutes, may realistically be required, confounding the tractability of running the simulation for a meaningful length of time at the population level (such as 1,000 generations, for example). However, discrete GRN models such as Boolean networks or discrete Bayesian networks cannot represent these sorts of sensory networks themselves. GRNs representing memory-based motifs used for cell-fate determination as previously described, however, may be more suitable as they could guide differentiation events at the individual-based level. These could act as switches to ensure that individuals change from one life cycle stage to another, and thus would therefore have important implications to the fitness of an individual.

### 4.6. Applications to Plant Science

As previously discussed, selection of individuals for certain traits may occur from a number of different selection regimes. In some populations, it may be that edaphic or other climactic effects such as light, as in the case of flowering time, or selection may be facilitated more from biotic interaction arising from pests or predators. Another example of a selection regime upon plants is crop domestication, a topic of considerable debate [146], where selection is imposed upon populations of crops by humans, who provide the biotic interaction. Interestingly, the nature of the human biotic interaction is so important that crop traits acquired through the domestication process are deleterious in nature. We believe that the described system of coupling GRNs with SIBMs is equally applicable to modeling selection as imposed by human cultivators as modeling selection by the wild. This is an ongoing research effort within our group. For modeling domestication, however, specific models may be required for simulating cultivator involvement, such as harvesting and sowing of crops, and removal of pests, for example.

#### 4.6.1. Domestication as a Selection Regime

Domestication represents an important model of evolution where all aforementioned levels of regulation played a role, including the interactions at the genic level, the population level and the roles of abiotic and biotic factors (such as local climactic effects on crops and the roles of weeds and pests to crop yield). Through domestication our crops have developed traits that better serve human needs in agriculture. These traits include the non-shattering phenotype within cereals, where wind is insufficient to mediate dispersal of seeds from the ears and human intervention is necessary; increased seed size, which enables seeds to be sown deeper within the soil due to the larger endosperm, therefore preventing seeds from blowing away from the farmers field; a loss of hooks and awns, helping to prevent loss of seed from the field; and enhanced culinary chemistry, allowing superior food products to be produced (for reviews, see [71,100,147,148,149]). All of the aforementioned domestication traits are heavily relied upon today. It is understood that the non-shattering phenotype is a monogenic trait that occurs within double-recessive homozygotes, whereas the larger seed size phenotype is a polygenic trait [71]. Understanding how such genes interact and the evolutionary processes behind the selection of these traits is an area that warrants further study. Intra-annual variation has also played important roles in the domestication process, as crops were sown and harvested at certain times of the year, and some crops have since developed a lack of sensitivity to environmental cues for flowering or germination (hence a loss of dormancy amongst seeds). A meta-analysis conducted by Munguía-Rosa *et al. *found that flowering time is still under selection in many plants [150] and increased fitness amongst populations has been seen to be associated with local alleles of flowering time in *Arabidopsis lyrata* [151]. A model for the simulation of vernalization in onion has been developed by Streck [152] which demonstrated a response in flowering to the temperature and to the duration of vernalization (in days), using statistical functions. However, this simulation was not at a population or a genetic level. Developing models that can incorporate a landscape genetics element and a GRN element could greatly improve our understanding on such phenotypic variation. Dormancy and germination are other complex plant-processes where regulation exists on a population genetics level, where periods of dormancy will have important effects on the emergent seedlings fitness, and where regulation exists at a systems-based level. Dormancy has been described as having a number of categories: morphological, physiological deep, physiological non-deep and physical dormancy [153]. Morphological dormancy arises due to an underdeveloped seed embryo that requires time to mature, whereas physical dormancy involves the development of a water impermeable seed coat that requires scarification. Physiological dormancy, however, arises due to an imbalance in the ratio of abscissic acid and giberrellins, with abscissic acid promoting dormancy [154]. Moisture and temperature (specifically thermoinhibition) are important environmental conditions that may induce germination and hydrothermal models have been developed (including one from Watt *et al. *[155]) for simulation of germination at different environmental conditions. These models lack the population, landscape and genetic elements to selection, however, which could be simulated with the use of SIBMs and incorporated GRNs.

#### 4.6.2. Simulation Models Accounting for Polyploidy amongst Plants

It is not uncommon for flowering plants to exhibit polyploidy [156]. Examples of triploid plants are apple and banana, tetraploids include durum and cotton, and bread wheat is an example of a hexaploid. Polyploidy of many flowering plants are relatively recent events whereas some flowering plants, such as tetraploid brassicas, are paleopolyploids after ancient genome duplication events [157]. Simulation models that simulate independent assortment of chromosomes may not be able to accurately reflect the gametogenesis of allopolyploids, as there is a tendency there for homoeologous chromosomes to preferentially pair during meiosis. However, a recent simulation model of meiosis developed by Voorips and Maliepard [158], called PedigreeSim, allows varying degrees of preferential pairing and the formation of different quadrivalent chromosomal configurations, which can be used for the study of allotetraploids. Future simulation studies will have to take into account similar approaches if polyploid plants or other organisms are to be accurately simulated.

#### 4.6.3. Functional-Structural Plant Modeling and Efforts in the Simulation of Plant Growth and Morphology

Understanding plant growth habit and morphology is of particular importance to agronomic and ecological studies, as plants react to their environment by adjusting their growth and morphology to maximize their gained benefits from nutrient acquisition. Thus modeling efforts that take plant growth and morphology according to simulated environmental conditions could be useful for determining the impact of changes to the availability of light, temperature or moisture, *etc*. A currently developing field within the plant science and computational biology disciplines is the field of functional-structural plant modeling (FSPM) [159,160,161]. Modeling efforts within this field are concerned with the acquisition of nutrients from sources such as light, carbon, water and soil minerals and how this impacts upon the growth and morphology of the resulting plants. Complex plant architectures comprising organs such as stalks, leaves and meristems are simulated, often in three dimensions, which take on mass and form complex morphologies. Widely-used algorithmic concepts behind these models are fractal-like rewriting systems called L-systems [162], where in the case of plants, the plant architecture is represented by a text string of components (or phytomers) which represent building blocks that comprise the plant, such as the stalks, branches, flowers and meristems. This systematic approach enables virtual plants to be simulated with realistic morphologies that grow and develop new morphologies over time. Such studies have been used to simulate leaf development according to light input in *Arabidopsis thaliana *[163], carbon-water acquisition in orange trees [164], carbon and nitrogen acquisition [165] and light competition [166] in general virtual plants, and hormone biosynthesis and photosynthetate of Poplar [167]; where graph-rewriting systems called relational growth grammars (RGGs) [168] based on L-systems were used to model a metabolic regulatory network to simulate biosynthesis. The aforementioned studies do not attempt to simulate the population genetics of these plants. However, a notable study by Buck-Sorlin *et al.* developed a FSPM of barley using RGGs, where a GRN of seven genes was used to synthesize giberrellic acid, which played a role in the growth and morphology of the virtual barley plants [169]. The genes were able to crossover, therefore sexual reproduction was simulated, allowing the resulting genotypes to influence the resultant barley phenotypes. Only five individuals were simulated per generation, however. A follow-up study used simulated rice morphologies, and the model was parameterized with quantitative trait loci taken from a cultivated population, allowing the phenotypic effects of the morphologies to be influenced by the input genotypes [170]. Another more recent rice FSPM study simulated growth rates and was parameterized with different genotypes, with different effects towards the growth rate [171]. These studies represent important modeling efforts with application towards G × E interactions. Bornhofen *et al. *[172] provided an interesting FSPM study that utilized an evolutionary L-Systems approach that allowed plant strategies to evolve. Their simulations began with distribution of 1,000 seed individuals throughout a heterogeneous environment (consisting of five patches) that grew into mature virtual plants according to procurement of biomass from the surrounding environment. Their individuals contained a mutating genotype that comprised a set of parameters involved with the life history of the individuals, their dispersal and the system rules concerned with biomass acquisition and distribution. The individuals were able to reproduce asexually. Interdisciplinary work involving FSPM and evolutionary biology or landscape genetics is an interesting avenue of research, although due to the scale required by landscape genetics studies it could be that computational costs involved may impede development of such models at present, as is a limitation discussed by Bornhofen. However, assuming that computational power increases in the future, larger populations of simulated plants within FSPM studies may provide an interesting insight into the demography and adaptation of a population according to nutrient resource availability. They may also provide an important way to study biotic interactions from competitors such as weeds.

## 5. Conclusions

In this review we have covered a number of aspects from the need for plant genetic modeling and simulation, concepts in population and landscape genetics and network motifs and inference within the field of systems biology. We believe that modeling systems that could incorporate regulation at the genic, genome, individual, population and environmental scales would be able to provide flexible systems for studying adaptation within highly dynamical environments. Population genetics simulations are often based upon simplifying assumptions that may not necessarily represent the complexity within a real population. Conversely, the complexities within large inferred GRNs may often be to such a degree that the noise arising from the numerous nodes may add ambiguity to each of their roles. By merging the two modeling systems, both could complement each other, as the complexity of large GRNs could be culled to the minimum set of nodes that is necessary to represent the system (obtaining this set could be another application of such models), and the GRNs at the population genetic level will provide an interface for interaction of genetic material with the environment. We are not describing an unnecessarily complex modeling approach intended to accurately represent nature in all its detail. Instead we are describing a general approach containing the requisite components to be able to simulate adaptation of individuals in certain environments through networks of interacting genes. The genes stimulate or repress each other according to their input and this invokes expression of a phenotype. This system provides greater dynamicity regarding simulation of phenotypic expression than by simply simulating QTLs when we consider the role of mutation to the interacting nodes, and therefore requires a dynamic environment, such as seen throughout the year. Such a model immediately has application to the simulation of time-dependent processes such as germination or flowering. Furthermore by coupling GRNs to SIBMs and allowing GRN evolution, through network rewiring or duplication, for example, we will be given an insight into how networks evolve as populations expand throughout a landscape, a field that to our knowledge remains largely unexplored. Simulation of movement of gene networks through heterogenous landscapes combined with stochastic evolutionary forces such as gene flow, mutation and genetic drift will allow emergent properties of GRN evolution and phenotypic diversity to be observed. Such observations may be difficult to achieve without a unified simulation model. This way we envision an approach to modeling GRN evolution that incorporates all levels of biological organization.
